# Mitochondrial Genome of the Indo‐Pacific Mesophotic Coral *Leptoseris columna* (Scleractinia: Agariciidae) Assembled Using PacBio Long‐Read Sequencing

**DOI:** 10.1002/ece3.73585

**Published:** 2026-06-09

**Authors:** Nomita Rani Adhikary, Daniel J. Barshis, J. Antonio Baeza

**Affiliations:** ^1^ Department of Fisheries Biology and Genetics Sher‐e‐Bangla Agricultural University Dhaka Bangladesh; ^2^ Department of Biological Sciences Old Dominion University Norfolk Virginia USA; ^3^ Department of Biological Sciences Clemson University Clemson South Carolina USA; ^4^ Departamento de Biología Marina Universidad Catolica del Norte Coquimbo Chile; ^5^ Smithsonian Marine Station at Fort Pierce Smithsonian Institution Fort Pierce Florida USA

**Keywords:** cryptic species, group I intron, hexacoral, mesophotic reefs, third generation sequencing

## Abstract

*Leptoseris columna*, a mesophotic coral species belonging to the family Agariciidae, is distributed throughout the Indo‐Pacific region. This species is considered as of “Least concern” by the IUCN, yet, faces multiple local and global stressors. To support conservation plans, this study sequenced and characterized the complete mitochondrial genome of *L. columna* . The complete mitochondrial genome of *Leptoseris columna* was assembled using PacBio long‐reads with a coverage of 459× per bp. The AT‐rich mitochondrial genome of *Leptoseris columna* is 18,546 bp long and comprises 13 protein‐coding genes (PCGs), 2 transfer RNA genes (trnM and trnW), and 2 ribosomal RNA genes, all located on the heavy strand. The *nad5* gene contains a long (11,235 bp) Group I intron. In the mitochondrial PCGs, TTT (Phe), TTA (Leu) and GTT (Val) codons were most abundant and a preference for synonymous codons ending in A or T, contributes to the AT‐rich nature of the studied mitogenome. The two tRNAs exhibited typical cloverleaf secondary structures. The assembled mitogenome also comprised two relatively long non‐coding regions (1031 and 591 bp) with numerous microsatellites and short tandem repeats. A Maximum Likelihood (ML) phylomitogenomic analysis based on 13 protein‐coding genes confirmed the monophyletic status of the order Scleractinia and family Agariciidae. However, the genus *Leptoseris* is polyphyletic. Other than supporting conservation plans, the newly assembled complete mitochondrial genome of *Leptoseris columna* will contribute to the advancing of our understanding of mesophotic coral systematics.

## Graphical abstract

The complete mitochondrial genome of *Leptoseris columna* was assembled using PacBio long read sequencing.

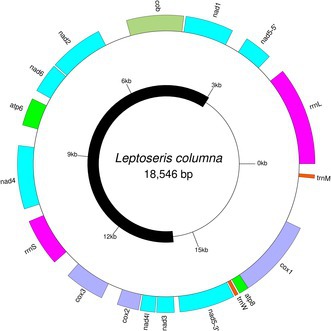

## 1 Introduction

Among scleractinian corals (order Scleractinia), members of the family Agariciidae dominate mesophotic reef communities (Bak et al. 2005), and in the aforementioned family, the genus *Leptoseris* comprises 18 species commonly inhabiting the lower mesophotic community down to depths of approx. 80 m (Englebert et al. 2017; Kahng and Kelley 2007). *Leptoseris* spp. have miniature tentacles and sparse corallites, which support efficient inorganic carbon and nitrogen exchange and improve assimilation of autotrophic nutrients (Ferrier‐Pagès et al. 2022).

In the genus *Leptoseris, L* *. scabra* has a wide geographic distribution in the Indo‐Pacific region; it can be found in the Red Sea, Southwestern Indian Ocean, Western Australia, the Hawaiian Archipelago in the North Pacific Ocean, Rapa Nui island in the South Pacific Ocean, and as far north as Kota Kinabalu in the South China Sea (Veron and Marsh 1988; Waheed and Hoeksema 2014; Kahng et al. 2017; Terraneo et al. 2017; Riegl et al. 2019; Hoarau et al. 2021; Sellanes et al. 2021; Gijsbers et al. 2023). *Leptoseris scabra* has been reported to exhibit remarkable corallum and corallite disparity and grows under many different conditions; in habitats dominated by soft sediments and hard rock, it is present in environments with (relatively) intense light exposure or in dark caves, and can be established both in clear and sediment‐rich waters (Dinesen 1980). The aforementioned morphological plasticity and habitat range suggest that this species may represent a cryptic species complex, and in line with this notion, a recent study based on nuclear single polymorphic variants reported highly genetically‐divergent lineages in this species (Gijsbers et al. 2023).

Like other hexacorals, *L. scabra* is exposed to numerous local (i.e., habitat modification, destruction and pollution) and global (i.e., climate change) stressors (Kleypas et al. 1999; Baker et al. 2008; Pandolfi et al. 2011). In particular, *L. scabra* populations have been negatively impacted by increased sedimentation and urban development (Waheed and Hoeksema 2014) as well as cyanobacteria and green algal blooms (Sellanes et al. 2021). Although *Leptoseris scabra* is currently classified as of “Least Concern” by the International Union for Conservation of Nature (IUCN) Red List, its current population trend is considered as decreasing (Cowburn et al. 2024).

To support biomonitoring and the formal future recognition of cryptic species of corals inhabiting mesophotic reefs in the Indo‐Pacific Ocean, in this study, we have performed a focused assembly of the mitogenome of a divergent lineage of *L. scabra* using genome sequence data previously used to assemble the nuclear genome of the studied specimen (Radice et al. 2025). The specimen we have sampled and from which we have obtained genomic data fits the description of *L. columna* (Yabe and Sugiyama, 1941), currently considered a synonym of *L. scabra* (Sheppard, 1987).

In this study, we have described in detail the mitochondrial genome of *Leptoseris columna*. Following recommendations in Baeza (2022) for the detailed analysis of mitochondrial genomes, we analyzed mitochondrial gene organization and nucleotide composition of the entire mitochondrial genome. We also estimated codon usage and relative synonymous codon usage of mitochondrial protein‐coding genes. Additionally, other than detecting tRNA genes in the studied mitochondrial genome, we predicted their secondary structures. We also conducted a detailed analysis of the non‐coding putative control region(s). Lastly, we evaluated the phylogenetic position of *L. columna* based on PCGs among species from the family Agariciidae. This newly assembled mitochondrial genome represents a new genomic resource to continue improving the understanding of the evolution of mesophotic coral reefs and will support evidence‐based conservation management in the studied species.

## 2 Materials and Methods

### 2.1 Coral Collection, DNA Extraction, Sequencing, and Mitochondrial Genome Assembly

Small sections from an adult coral of *Leptoseris columna* were collected by divers using closed circuit rebreathers during March 2nd, 2022, from 37.3 m depth, while exploring the forereef slope off Leone village (14.342° S, 170.789° W), Tutuila Island, American Sāmoa (Radice et al. 2025), under permission granted by the American Sāmoa Department of Marine and Wildlife Resources (permit #2020/004). The collected coral fragments were transported to the laboratory and maintained at high temperatures (~30°C–31°C) for more than a week to decrease the concentration of symbiotic dinoflagellates. Then, all “bleached” coral fragments were preserved in DNA/RNA Shield buffer (Zymo) and frozen at −80°C until genomic DNA (gDNA) was extracted. Physical voucher specimens are held at the Natural History Museum of Old Dominion University, Norfolk, Virginia, USA. The protocols for genomic DNA extraction, Pacific Biosciences (PacBio) library preparation, and sequencing were used without modification as described by Radice et al. (2025). In short, Genomic DNA was extracted from preserved samples and purified using ethanol precipitation, sodium acetate, and phenol–chloroform–isoamyl alcohol extraction to remove contaminants. DNA concentration and purity were assessed using fluorometric and Nanodrop assay methods, and molecular weight was measured using TapeStation genomic tape and Pulse Field gel electrophoresis (PFGE). A PacBio SMRTbell library (~20 kb) was constructed using SMRTbell Express Template Prep Kit 2.0 (PacBio, USA) following the manufacturer's protocol. The library was then sequenced on an PacBio Sequel platform. A total of 1,749,817 sequences (one lane with sequence length range: 103–39,822, GC content = 38%, 38.22 Gbp) made available by the sequencing facility were used for the genome assembly of *L. columna* with the software MITOHiFi (Uliano‐Silva et al. 2023) and the mitochondrial genome of the congeneric *L. papyracea* (GenBank accession number: LC792539) that was used as a seed.

### 2.2 Mitochondrial Genome Annotation and Analysis

The assembled mitogenome of *Leptoseris columna* was annotated using the software MITOS2 (Donath et al. 2019) as implemented in the web platform Galaxy (The Galaxy Community 2024) with the invertebrate genetic code. Manual curation of the *in silico* annotation was conducted with the aid of the online translation tool ExPASy translate (https://web.expasy.org/translate/—SIB Swiss Institute of Bioinformatics (Artimo et al. 2012)) and the software MEGA12 (Kumar et al. 2024). The complete circular mitochondrial genome was visualized using the program GenomeVx (Conant and Wolfe 2008). The nucleotide usage analysis of the complete mitochondrial genome of *Leptoseris columna* species was computed in the software MEGA12 (Kumar et al. 2024). Codon usage for each protein‐coding gene was determined in the web server Codon Usage (https://www.bioinformatics.org/sms2/codon_usage.html—Stothard 2000) with default parameters and using the invertebrate mitochondrial code. Relative Synonymous Codon Usage (RSCU) was calculated using EZ Codon on the EZmito web server (http://ezmito.unisi.it/ezcodon), with values representing the ratio of observed to expected synonymous codon frequencies for each amino acid (Lee 2018; Cucini et al. 2021).

The secondary structures of tRNA genes were predicted using the software MiTFi, which is embedded within the MITOS2 pipeline (Donath et al. 2019) and visualized using the web server Forna (http://rna.tbi.univie.ac.at/forna/) (Kerpedjiev et al. 2015).

Lastly, microsatellites and tandem repeats present in the longest non‐coding regions of the studied mitochondrial genome were discovered and identified using the web servers Microsatellite Repeats Finder (http://insilico.ehu.es/mini_tools/microsatellites/—Bikandi et al. 2004) and Tandem Repeat Finder (https://tandem.bu.edu/trf/trf.basic.submit.html—Benson 1999), respectively. The secondary structure of the non‐coding regions were predicted using the web server RNa‐structure (https://rna.urmc.rochester.edu/RNastructureWeb—Bellaousov et al. 2013).

### 2.3 Phylogenetic Placement of *Leptoseris columna*

The phylogeny of scleractinian hexacorals, including *L. columna* , was inferred based on 13 mitochondrial protein‐coding genes using Maximum Likelihood (ML) analysis. The analysis included the newly assembled and annotated mitochondrial genome of *Leptoseris columna* plus eight mitochondrial genomes from species belonging to the family Agariciidae, together with 116 complete mitochondrial genomes available in GenBank representing 19 other scleractinian families. Mitochondrial genomes from ten species belonging to the order Corallimorpharia were included as outgroups. The Maximum Likelihood (ML) analysis was conducted using the program PhyloSuite v2 (Zhang et al. 2020; Zhao et al. 2025). The 13 mitochondrial protein‐coding genes were aligned using the program MAFFT (Katoh and Standley 2013; Zhang et al. 2020). Following alignments, GBlocks was used to trim the poorly aligned sequences using the default setting (Castresana 2000). Subsequently, the 13 protein‐coding genes were concatenated into a single file partitioned by PCG and the best fitting models (GTR, GTR + G) of nucleotide evolution (Table S1) were selected using the greedy algorithm and AICc model selection criterion in the program PartitionFinder2 (Lanfear et al. 2017; Zhang et al. 2020). The Maximum likelihood analysis was conducted using IQ‐TREE with default settings (Nguyen et al. 2015; Zhang et al. 2020). The robustness of the maximum‐likelihood tree topology was assessed using 1000 ultrafast bootstrap (UFBoot) iterations. Finally, the phylogenetic tree was visualized using the software FigTree v1.4.4 (https://tree.bio.ed.ac.uk/software/figtree/—Rambaut and Drummond 2018).

## 3 Results and Discussion

**FIGURE 1:**
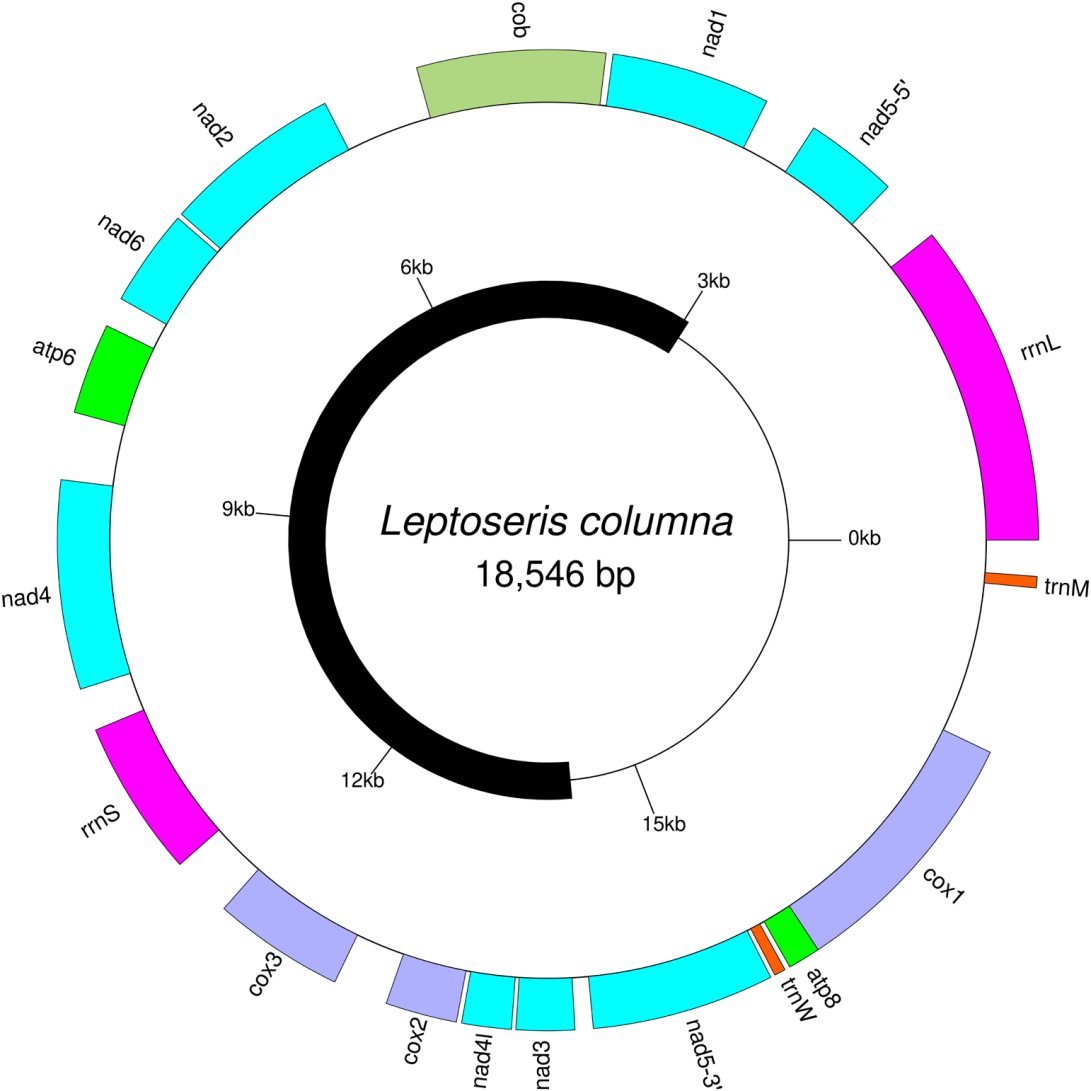
Mitochondrial genome visualization of *Leptoseris columna*.

The software MitoHiFi assembled and circularized the mitochondrial genome of *Leptoseris columna* with a coverage (per nucleotide) of 459× (GenBank accession number: PX841739). The length of the complete mitogenome of *Leptoseris columna* is 18,546 bp and codes for 13 protein‐coding genes (PCGs), 2 transfer RNA genes (trnM and trnW), and 2 ribosomal RNA genes (Figure 1). The mitochondrial gene order of *Leptoseris columna* conforms to the canonical scleractinian Type II arrangement (Lin et al. 2014; Medina et al. 2006) and is identical to that observed in other agariciid corals ( *Pavona decussata* —Shi et al. 2016; *Agaricia humilis* and *A. fragilis* —Wares 2014; *Pseudomadrepora carolina, Thalamophyllia gasti*, and *Thalamophyllia riisei* —Vaga et al. 2025). The newly assembled mitochondrial genome also includes two relatively long non‐coding regions measuring 1031 bp and 591 bp, and a long Group I intron (11,235 bp) that bisected the *nad5* gene and encompassed 10 PCGs and rrnS in *Leptoseris columna*. In previous studies, a group I intron was reported to bisect the mitochondrial *nad5* gene ( *M. myriaster* —Tucker et al. 2023; *Pocillopora meandrina* —Fu and Hong 2025; *Diploastrea heliopora* —Xiao et al. 2021; Fukami and Knowlton 2005) and, in some species, the *cox1* gene ( *Seriatopora caliendrum* and *Seriatopora hystrix* —Chen, Chiou, et al. 2008; *Diploastrea heliopora* —Xiao et al. 2021). Overall, Group I introns in mitochondrial genomes are regarded as functional and an evolutionarily conserved feature in hexacorals (Medina et al. 2006; Lin et al. 2014).

The positive strand of the *Leptoseris columna* mitochondrial genome has a nucleotide composition of A = 23.8%, G = 25.4%, C = 15.4%, and T = 35.0%, resulting in a 59.2% A + T content. Agariciidae corals generally exhibit AT‐rich mitochondrial genomes, ranging from 59.2% in *L. columna* , *L. papyracea* (LC792539.1), and *Pavona decussata* (KP231535.1) (Shi et al. 2016) to 60.1% in *Thalamophyllia riisei* (PV815617.1; Vaga et al. 2025). *Leptoseris columna* shows a negative AT‐skew (−0.196) and positive GC‐skew (0.245), with Thymine as the most abundant and Cytosine as the least abundant nucleotide. All cofamilial species have a negative AT‐skew (highest −0.187 in *Thalamophyllia gasti* PV815622.1 and *Agaricia humilis* DQ643831.1; lowest −0.196 in *L. columna* , *L. papyracea* , and *P. decussata* ) and a consistently positive GC‐skew (highest 0.25 in *Pseudomadrepora carolina*; lowest 0.237 in *A. humilis* ) (Vaga et al. 2025; Medina et al. 2006; Hisata et al. 2025; Shi et al. 2016). The observed biases likely result from asymmetric mutational constraints (Hassanin et al. 2005) and DNA replication processes that increase AT mitochondrial content (Perna and Kocher 1995).

Among the mitochondrial PCGs, seven PCGs (*nad5, nad1, nad2, nad6, nad4, cox3, nad4l*) start with ATA start codons, whereas the other five PCGs (*cob, atp6, cox2, nad3, cox1*) start with ATG codons (Table 1). In most mitochondrial protein‐coding genes, ATG or ATA function as the start codon. Also, in *L. columna* , the *atp8* gene starts with the non‐canonical codon GTG, in agreement with that observed in a few other coral mitochondrial genomes (Fu and Hong 2025; Kayal et al. 2012). Additionally, most of the PCGs (*N* = 8; *nad1, nad2, nad6, cox2, nad4l, nad3, atp8, cox1*) end with TAA codons, whereas five PCGs (*cob, atp6, nad4, cox3, nad5*) terminate with TAG codons (Table 1). All protein‐coding genes contain complete stop codons, either TAA or TAG (Table 1).

**TABLE 1:** Mitochondrial genome of *Leptoseris columna*: Arrangement and annotation.

Gene name	Start	Stop	Strand	Length (bp)	Start codon	Stop codon	Anti codon	Cont.
rrnL	1	1984	+	1984				218
nad5‐5′	2375	2947	+	573	ATA			390
nad1	3272	4243	+	972	ATA	TAA		324
cob	4286	5437	+	1152	ATG	TAG		42
nad2	6029	7126	+	1098	ATA	TAA		591
nad6	7158	7753	+	596	ATA	TAA		31
atp6	7937	8494	+	558	ATG	TAG		183
nad4	8907	10,184	+	1278	ATA	TAG		412
rrnS	10,446	11,405	+	960				261
cox3	11,780	12,583	+	804	ATA	TAG		374
cox2	12,918	13,355	+	438	ATG	TAA		334
nad4l	13,387	13,689	+	303	ATA	TAA		31
nad3	13,716	14,072	+	357	ATG	TAA		26
nad5‐3′	14,183	15,301	+	1119		TAG		110
trnW	15,329	15,398	+	70			tca	27
atp8	15,431	15,649	+	219	GTG	TAA		32
cox1	15,631	17,226	+	1596	ATG	TAA		−19
trnM	18,258	18,328	+	71			cat	1031

**FIGURE 2:**
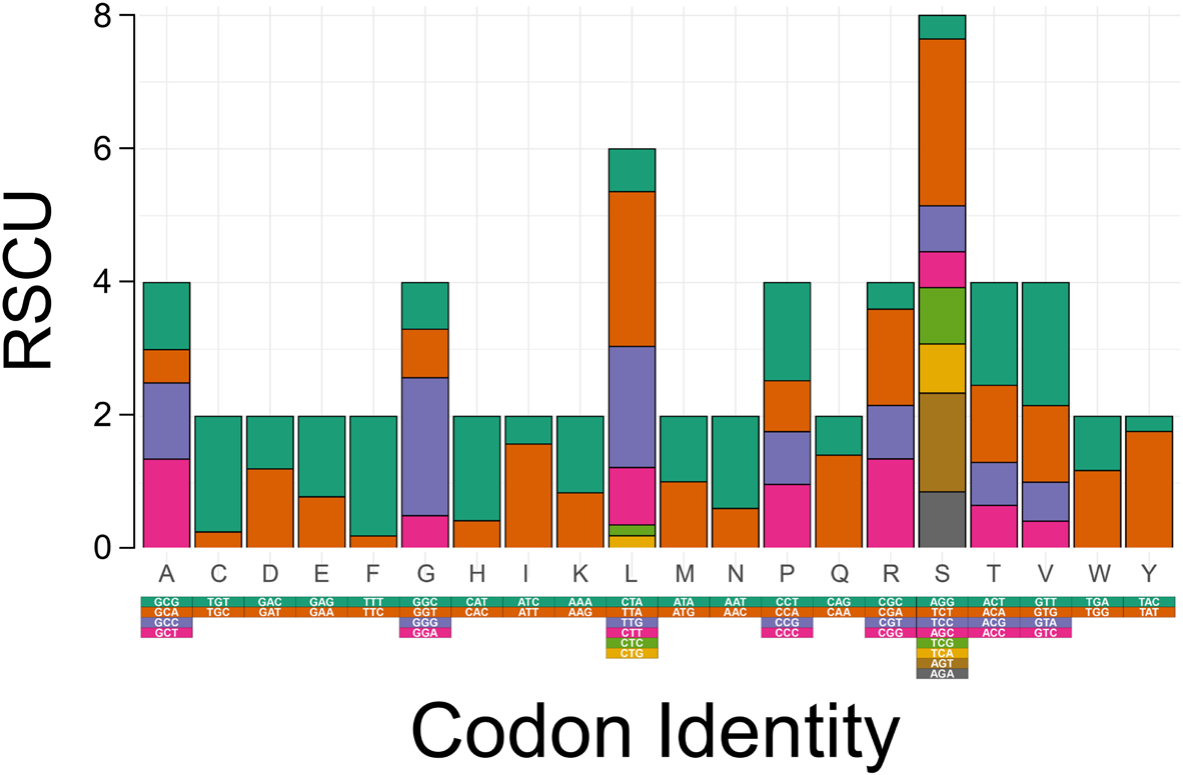
Relative synonymous codon usage (RSCU) analysis of 13 mitochondrial protein‐coding genes in *L. columna*.

Codon usage of the protein‐coding genes in the mitochondrial genome of *Leptoseris columna* is uneven. The most abundantly used codons were all A + T‐rich, including TTT (Phe, 282 occurrences; 7.53%), TTA (Leu, 216 occurrences; 5.77%), and GTT (Val, 171 occurrences; 4.57%). Conversely, excluding stop codons, the least utilized codons were G + C‐rich: TGC (Cys, 5 occurrences; 0.13%), CGC (Arg, 5 occurrences; 0.13%), and AGG (Ser, 14 occurrences; 0.37%) (Table S2). Among the protein‐coding genes of the analyzed mitochondrial genome, the amino acid serine (S) was encoded by the highest number of synonymous codons (eight), followed by leucine (L) with six. The majority of the remaining amino acids were encoded by two or four synonymous codons, with two‐codon encoding being more prevalent (Figure 2). Synonymous codons ending with adenine (A) or thymine (T) at the third codon position were preferentially used, showing a higher average RSCU value (1.22 ± 0.52) compared with synonymous codons ending in guanine (G) or cytosine (C), which exhibited a lower average RSCU (0.77 ± 0.46) (Table S3). This pattern reflects the AT bias typical of hexacoral mitochondrial genomes, particularly thymine enrichment at the first and second codon positions of protein‐coding genes (Kitahara et al. 2014).

**FIGURE 3:**
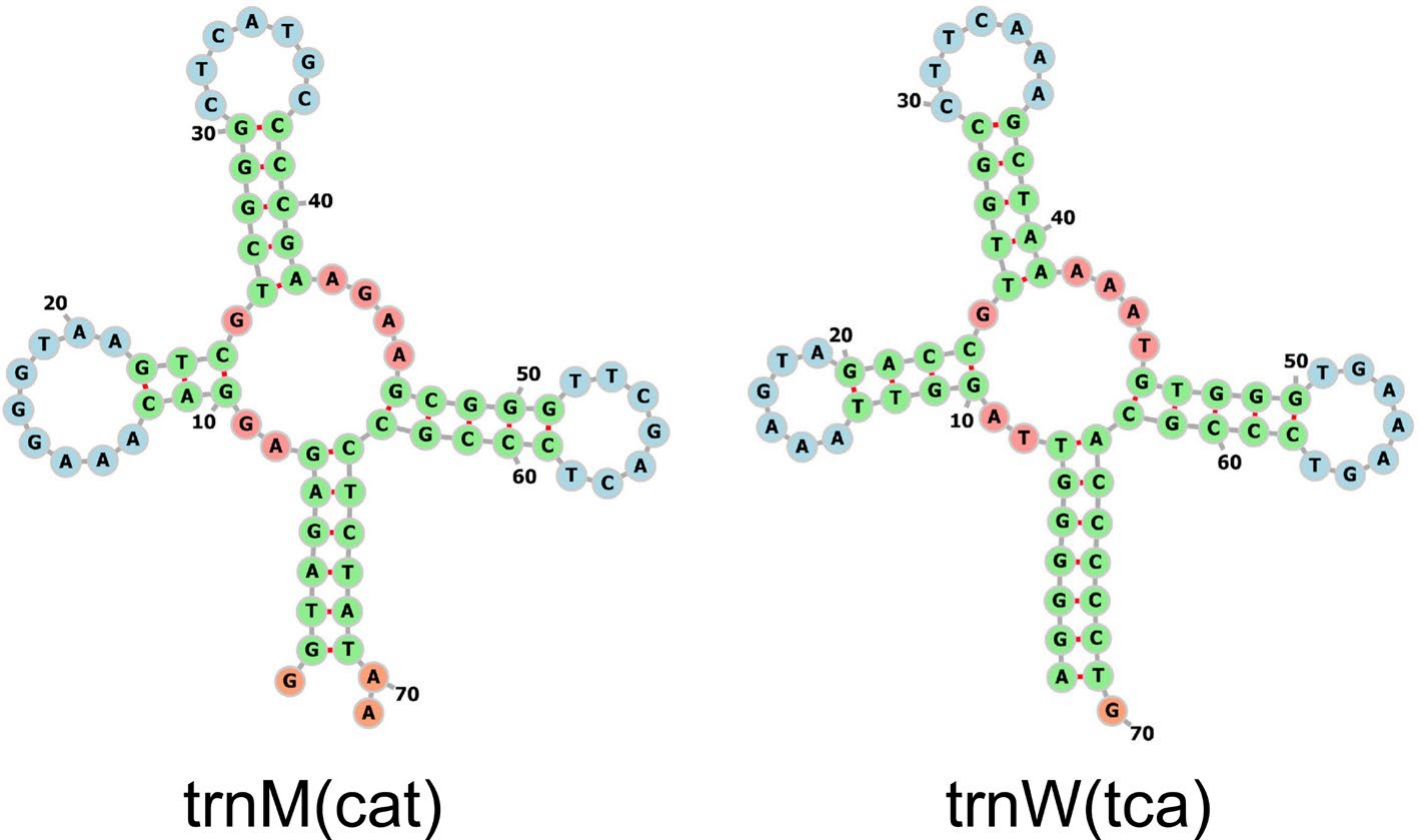
Mitochondrial tRNAs secondary structure in *L. columna*.

The mitochondrial genome of *Leptoseris columna* contains two transfer RNA (tRNA) genes, tRNA‐tryptophan (trnW) that is flanked by nad5‐3′ and *atp8*, and tRNA‐methionine (trnM), positioned between *cox1* and rrnL (Figure 1). Both tRNAs show typical cloverleaf secondary structure, consisting of the amino acid acceptor stem, the anticodon loop, the TψC loop, the D loop, and the variable (V) region (Figure 3). The small number of mitochondrial‐encoded tRNA genes is a general feature of Scleractinian corals and other representatives of the phylum Cnidaria (Novosolov et al. 2022). Agariciids (e.g., *Pavona decussata* : Shi et al. 2016; *Madrepora carolina* : Vaga et al. 2025) also encode two tRNAs, although some scleractinian families exhibit a duplicated trnW (Pocilloporidae—Chen, Dai, et al. 2008; Chen, Chiou, et al. 2008; Flot et al. 2008) or single trnM (Isididae ‐ Brugler and France 2008).

The complete mitochondrial genome of *Leptoseris columna* contains two rRNAs, the small rRNA and the large rRNA located on the heavy strand. The small rRNA is positioned between the *nad4* and *cox3* genes while the large rRNA is located between trnM and *nad5*. The overall nucleotide composition of the rrnL gene is characterized by A = 32.5%, T = 26.7%, G = 25.9%, and C = 15.0%, and the rrnS gene comprises A = 31.1%, T = 28.1%, G = 25.0%, and C = 15.7%. The two ribosomal rRNA genes are A + T rich.

The two relatively large non‐coding regions in the mitochondrial genome of *Leptoseris columna* were 1031 and 591 bp long, flanked by *cox1* and trnM(cat), and *cob* and *nad2*, respectively (Figure 1, Table 1). The A + T content of the shorter 591 bp non‐coding region was similar (A + T content = 59.9%) to that estimated for the complete mitochondrial genome (59.2%), while the longer 1031 bp non‐coding region has a notably lower A + T content (48.6%) than that observed for the entire mitochondrial genome. In the long and short non‐coding regions, a total of 11 and 7 microsatellite repeats were detected, respectively (Tables S4 and S6). All microsatellites comprised dinucleotide motifs repeated between 3 and 5 times, and the great majority were TG‐rich (Tables S4 and S6). In the longest 1031 bp non‐coding region, two tandem repeats were detected. The first one is 32 bp in length (motif: 5′‐AGT TTT GTG TGG GAG GGG TCA AGG TTC CCT CG‐3′), repeated three times (Table S5, Tandem Repeat 1), and the second is 41 bp long (motif: 5′ GGG GTC CCC TCG ACT TTT GTG GAG GGG ACC CCA AAG GAG GG‐3′), repeated 2 times (Table S5, Tandem Repeat 2). In the shorter 591 bp non‐coding region, a single tandem repeat, 69 bp in length, was detected (motif: 5′‐TGG CTG GTA CCC AAG TGA TGG TAG TGT TTT TTT TCC TGA AGC ATT TAT GGG CGA GTT GTG GTT TGA AAA‐3′), repeated two times (Table S7). Lastly, multiple stem and loop secondary structures were predicted along the entire length of the two non‐coding regions by the software RNaFold (Figures S1 and S2). 15 other non‐coding regions were also detected in the studied mitochondrial genome, all of them shorter than 412 bp, and none of them exhibited microsatellites and short tandem repeats.

**FIGURE 4:**
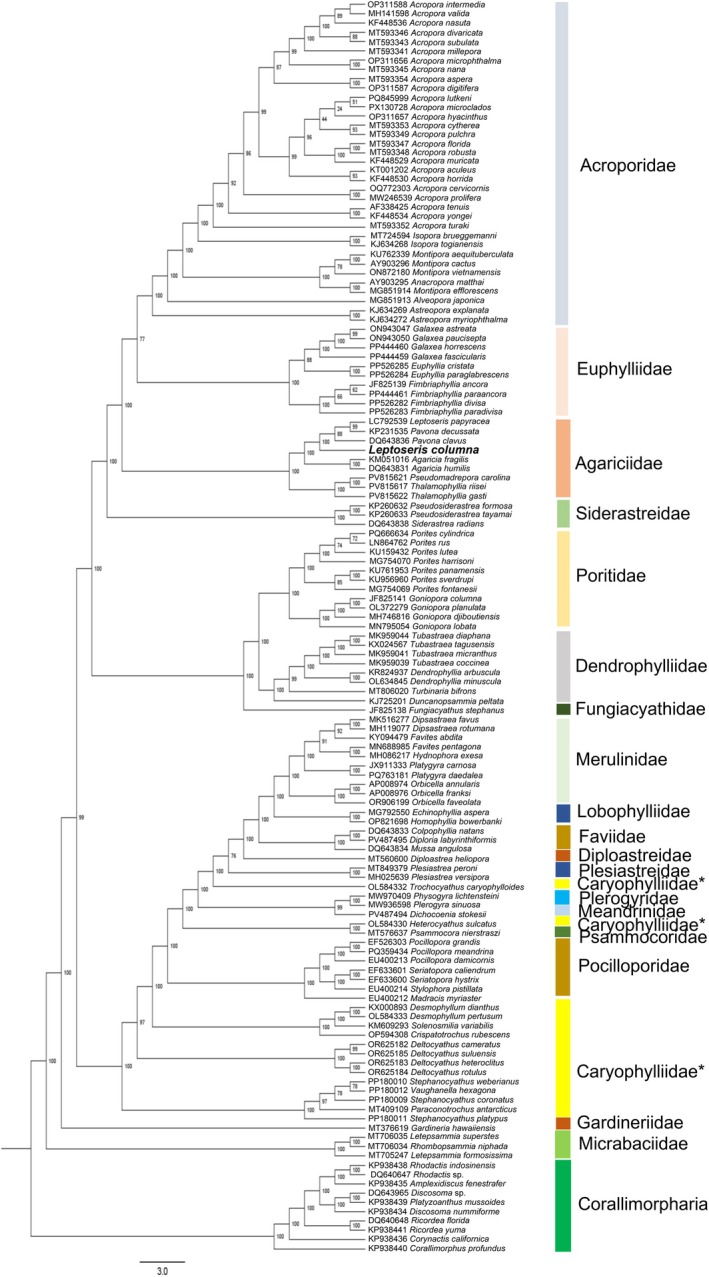
Phylomitogenomic analysis of *L. columna* along with related species in the order Scleractinia. The total evidence phylogenetic tree obtained from ML analysis based on a concatenated alignment of codons of the 13 protein‐coding genes present in the mitochondrial genome of *Leptoseris columna* and other representatives of the order Scleractinia. Ten additional mitochondrial genomes from the order Corallimorpharia were used as outgroups, including members from the family Discosomatidae (*n* = 6), Corallimorphidae (*n* = 2), and Ricordeidae (*n* = 2). Branch lengths are not included in the figure for clarity. Numbers displayed on the branches correspond to bootstrap support values.

The ML phylogenetic tree (135 terminals, 11,697 characters, 6663 informative sites) fully supported the monophyly of the order Scleractinia (bv = 100; Figure 4), with most interfamilial relationships in line with those reported in previous phylogenetic and phylogenomic studies (Tucker et al. 2023; Baeza and Rosales 2025; Seiblitz et al. 2020, 2022; Xiao et al. 2021; Vaga et al. 2022). Within the order Scleractinia, the monophyletic family Micrabaciidae (fully supported, bv = 100) occupied an early branching position; it was sister to another fully supported clade (bv = 100) containing all other species used in our analysis. Similarly, in the latter clade, *Gardinera hawaiiensis*, the single species belonging to the family Gardineriidae, was sister to another fully supported clade (bv = 100) containing all other species used in our analysis except representatives of the family Micrabaciidae. In the latter clade, our phylogenetic tree recovered two previously recognized major subclades within Scleractinia: the ‘robust’ and ‘complex’ subclades (Lin et al. 2011; Kitahara et al. 2010, 2016; Stolarski et al. 2011; Campoy et al. 2020; Seiblitz et al. 2020). In the ‘robust’ subclade, fully or well‐supported families comprised (in alphabetical order) Astrangiidae, Caryophylliidae, Diploastreidae, Faviidae, Lobophylliidae, Meruliniidae, Plerogyridae, Pocilloporidae, and Psammocoriidae. In the ‘complex’ subclade, fully or well‐supported families comprised (in alphabetical order) Agariciidae, Dendrophylliidae, Euphylliidae, Fungiacyathidae, Poritidae, and Siderastreidae. Importantly, the family Caryophylliidae in the robust subclade was polyphyletic, in agreement with previous phylogenetic analyses also based on the phylogenetic signal provided by mitochondrial PCGs (Tucker et al. 2023; Baeza and Rosales 2025).

Lastly, all species belonging to the family Agariciidae formed a fully supported monophyletic clade (bv = 100). However, *Leptoseris columna* did not cluster with *L. papyracea* (LC792539), the only congeneric species with a mitochondrial genome available in GenBank. Instead, *L. columna* clustered with *Pavona clavus* and *P. decussata* , and *L. papyracea* was sister to this latter clade (bv = 99), indicating that the genus *Leptoseris* is polyphyletic, as reported previously (Le Goff‐Vitry et al. 2004; Luck et al. 2013; Terraneo et al. 2017). We note that species belonging to the genera *Pavona* and *Leptoseris* have independently evolved similar macro‐morphological adaptations to the deep‐water environment, thus causing taxonomic incongruence between genetic evidence and traditional macromorphology‐based taxonomy (Luck et al. 2013). We recommend revisiting the taxonomy of these two genera using an integrative taxonomic approach (also, see Le Goff‐Vitry et al. 2004; Luck et al. 2013; Terraneo et al. 2017).

## 4 Conclusion

This study assembled the complete mitochondrial genome of *Leptoseris columna*, an ecologically relevant deep‐water reef‐forming coral species inhabiting the Indo‐Pacific region, which is increasingly threatened by anthropogenic pressures. The newly assembled mitogenome of *L. columna* represents a genomic resource that will help with biomonitoring using non‐intrusive strategies (environmental DNA) and advance our understanding of coral systematics.

## Author Contributions

**Nomita Rani Adhikary:** data curation (equal), formal analysis (equal), investigation (equal), methodology (equal), validation (equal), visualization (equal), writing – original draft (equal), writing – review and editing (equal). **Daniel J. Barshis:** data curation (equal), formal analysis (equal), funding acquisition (equal), resources (equal), supervision (equal), validation (equal), writing – original draft (equal), writing – review and editing (equal). **J. Antonio Baeza:** conceptualization (equal), data curation (equal), funding acquisition (equal), investigation (equal), methodology (equal), project administration (equal), resources (equal), supervision (equal), validation (equal), writing – original draft (equal), writing – review and editing (equal).

## Funding

This work was supported by Clemson University SUCCEED, 1.

## Conflicts of Interest

The authors declare no conflicts of interest.

## Supporting information


**Figure S1:** Secondary structure prediction (lowest free energy structure) in the long non‐coding regions (1031 bp) present in the mitochondrial genome of *Leptoseris columna*.
**Figure S2:** Secondary structure prediction (lowest free energy structure) in the shorter non‐coding regions (591 bp) present in the mitochondrial genome of *Leptoseris columna*.
**Table S1:** Optimal partition scheme and best fitting models of nucleotide evolution.
**Table S2:** Codon usage analysis.
**Table S3:** Relative Synonymous Codon Usage (RSCU) value analysis.
**Table S4:** Long non‐coding region: Microsatellite repeats.
**Table S5:** Tandem Repeats Finder (Long non‐coding region_1031bp): Tandem Repeat 1: Length: 1031: Found at i: 904 original size: 32 final size: 32.
**Table S6:** Shorter non‐coding region: Microsatellite repeats.
**Table S7:** Tandem Repeats Finder (Shorter non‐coding_591bp): Found at i:367 original size:69 final size:69.

## Data Availability

The mitochondrial genome described here is accessible in GenBank with accession number PX841739.
