# Supplementary material for: Mitochondrial Genome of the Indo‐Pacific Mesophotic Coral Leptoseris columna (Scleractinia: Agariciidae) Assembled Using PacBio Long‐Read Sequencing
Source: Ecol Evol. 2026 Jun 9;16(6):e73585. doi: 10.1002/ece3.73585 (PMC13247554; doi:10.1002/ece3.73585)
Supplement: Supplementary file 1 — Figure S1: Secondary structure prediction (lowest free energy structure) in the long non‐coding regions (1031 bp) present in the mitochondrial genome of Leptoseris columna. Figure S2: Secondary structure prediction (lowest free energy structure) in the shorter non‐coding regions (591 bp) present in the mitochondrial genome of Leptoseris columna. Table S1: Optimal partition scheme and best fitting models of nucleotide evolution. Table S2: Codon usage analysis. Table S3: Relative Synonymous Codon Usage (RSCU) value analysis. Table S4: Long non‐coding region: Microsatellite repeats. Table S5: Tandem Repeats Finder (Long non‐coding region_1031bp): Tandem Repeat 1: Length: 1031: Found at i: 904 original size: 32 final size: 32. Table S6: Shorter non‐coding region: Microsatellite repeats. Table S7: Tandem Repeats Finder (Shorter non‐coding_591bp): Found at i:367 original size:69 final size:69. [file ECE3-16-e73585-s001.docx]

**Mitochondrial genome of the Indo-Pacific mesophotic coral *Leptoseris columna***

**(Scleractinia: Agariciidae) assembled using PacBio long-read sequencing**

**Nomita Rani Adhikary 1, Daniel J. Barshis 2, J. Antonio Baeza 3,4,5**

1 Department of Fisheries Biology and Genetics, Sher-e-Bangla Agricultural University, Dhaka, Bangladesh

2 Department of Biological Sciences, Old Dominion University, Norfolk, VA, USA

3 Department of Biological Sciences, Clemson University, Clemson, SC, USA

4 Departamento de Biología Marina, Universidad Catolica del Norte, Coquimbo, Chile

5 Smithsonian Marine Station at Fort Pierce, Smithsonian Institution, Fort Pierce, FL, USA

**Supplementary Materials**

**Table S1.** Optimal partition scheme and best fitting models of nucleotide evolution

| **Subset partitions** | **Best model** |
| --- | --- |
| P1: (atp6, cox3, cytb) | GTR+G |
| P2: (nad4l) | GTR+G |
| P3: (nad1) | GTR+G |
| P4: (nad2, nad6) | GTR+G |
| P5: (nad3, nad4) | GTR+G |
| P6: (atp8) | GTR+G |
| P7: (nad5) | GTR+G |
| P8: (cox1) | GTR |
| P9: (cox2) | GTR+G |

**Table S2. Codon usage analysis:**

Codon Usage results

Amino Acid Codon Number /1000 Fraction

Ala GCG 68.00 18.18 0.25

Ala GCA 34.00 9.09 0.13

Ala GCT 92.00 24.59 0.34

Ala GCC 77.00 20.58 0.28

Cys TGT 32.00 8.55 0.86

Cys TGC 5.00 1.34 0.14

Asp GAT 51.00 13.63 0.61

Asp GAC 33.00 8.82 0.39

Glu GAG 65.00 17.38 0.60

Glu GAA 43.00 11.49 0.40

Phe TTT 282.00 75.38 0.90

Phe TTC 33.00 8.82 0.10

Gly GGG 165.00 44.11 0.52

Gly GGA 41.00 10.96 0.13

Gly GGT 58.00 15.50 0.18

Gly GGC 56.00 14.97 0.17

His CAT 61.00 16.31 0.78

His CAC 17.00 4.54 0.22

Ile ATT 157.00 41.97 0.79

Ile ATC 41.00 10.96 0.21

Lys AAG 41.00 10.96 0.43

Lys AAA 55.00 14.70 0.57

Leu TTG 169.00 45.18 0.30

Leu TTA 216.00 57.74 0.39

Leu CTG 20.00 5.35 0.04

Leu CTA 60.00 16.04 0.11

Leu CTT 80.00 21.38 0.14

Leu CTC 15.00 4.01 0.03

Met ATG 107.00 28.60 0.51

Met ATA 103.00 27.53 0.49

Asn AAT 60.00 16.04 0.69

Asn AAC 27.00 7.22 0.31

Pro CCG 29.00 7.75 0.20

Pro CCA 28.00 7.48 0.19

Pro CCT 54.00 14.43 0.37

Pro CCC 36.00 9.62 0.24

Gln CAG 21.00 5.61 0.29

Gln CAA 51.00 13.63 0.71

Arg CGG 17.00 4.54 0.34

Arg CGA 18.00 4.81 0.36

Arg CGT 10.00 2.67 0.20

Arg CGC 5.00 1.34 0.10

Ser AGG 14.00 3.74 0.04

Ser AGA 34.00 9.09 0.11

Ser AGT 58.00 15.50 0.18

Ser AGC 21.00 5.61 0.07

Ser TCG 33.00 8.82 0.11

Ser TCA 29.00 7.75 0.09

Ser TCT 98.00 26.20 0.31

Ser TCC 27.00 7.22 0.09

Thr ACG 25.00 6.68 0.16

Thr ACA 45.00 12.03 0.29

Thr ACT 60.00 16.04 0.38

Thr ACC 26.00 6.95 0.17

Val GTG 106.00 28.33 0.29

Val GTA 54.00 14.43 0.15

Val GTT 171.00 45.71 0.46

Val GTC 40.00 10.69 0.11

Trp TGG 57.00 15.24 0.59

Trp TGA 39.00 10.43 0.41

Tyr TAT 140.00 37.42 0.89

Tyr TAC 18.00 4.81 0.11

End TAG 5.00 1.34 0.38

End TAA 8.00 2.14 0.62

**Table S3.** Relative Synonymous Codon Usage (RSCU) value analysis**:**

| **Species** | **Codon** | **RSCU** | **AA** | **1st position** | | | | **2nd position** | | | | **3rd position** | | | |
| --- | --- | --- | --- | --- | --- | --- | --- | --- | --- | --- | --- | --- | --- | --- | --- |
|  |  |  |  | **A** | **T** | **C** | **G** | **A** | **T** | **C** | **G** | **A** | **T** | **C** | **G** |
| *L. scabra* | ATA | 0.98 | M | 0.98 |  |  |  |  | 0.98 |  |  | 0.98 |  |  |  |
| *L. scabra* | AAA | 1.15 | K | 1.15 |  |  |  | 1.15 |  |  |  | 1.15 |  |  |  |
| *L. scabra* | CTA | 0.64 | L |  |  | 0.64 |  |  | 0.64 |  |  | 0.64 |  |  |  |
| *L. scabra* | TGA | 0.81 | W |  | 0.81 |  |  |  |  |  | 0.81 | 0.81 |  |  |  |
| *L. scabra* | AGG | 0.36 | S | 0.36 |  |  |  |  |  |  | 0.36 |  |  |  | 0.36 |
| *L. scabra* | TTA | 2.31 | L |  | 2.31 |  |  |  | 2.31 |  |  | 2.31 |  |  |  |
| *L. scabra* | GAC | 0.79 | D |  |  |  | 0.79 | 0.79 |  |  |  |  |  | 0.79 |  |
| *L. scabra* | TCT | 2.50 | S |  | 2.50 |  |  |  |  | 2.50 |  |  | 2.50 |  |  |
| *L. scabra* | GAT | 1.21 | D |  |  |  | 1.21 | 1.21 |  |  |  |  | 1.21 |  |  |
| *L. scabra* | TTG | 1.81 | L |  | 1.81 |  |  |  | 1.81 |  |  |  |  |  | 1.81 |
| *L. scabra* | ACT | 1.54 | T | 1.54 |  |  |  |  |  | 1.54 |  |  | 1.54 |  |  |
| *L. scabra* | GTT | 1.84 | V |  |  |  | 1.84 |  | 1.84 |  |  |  | 1.84 |  |  |
| *L. scabra* | TAC | 0.23 | Y |  | 0.23 |  |  | 0.23 |  |  |  |  |  | 0.23 |  |
| *L. scabra* | TTT | 1.79 | F |  | 1.79 |  |  |  |  | 1.79 |  |  | 1.79 |  |  |
| *L. scabra* | GGC | 0.70 | G |  |  |  | 0.70 |  |  |  | 0.70 |  |  | 0.70 |  |
| *L. scabra* | CAG | 0.58 | Q |  |  | 0.58 |  | 0.58 |  |  |  |  |  |  | 0.58 |
| *L. scabra* | GGT | 0.73 | G |  |  |  | 0.73 |  |  |  | 0.73 |  | 0.73 |  |  |
| *L. scabra* | GCG | 1.00 | A |  |  |  | 1.00 |  |  | 1.00 |  |  |  |  | 1.00 |
| *L. scabra* | ATC | 0.41 | I | 0.41 |  |  |  |  | 0.41 |  |  |  |  | 0.41 |  |
| *L. scabra* | ATG | 1.02 | M | 1.02 |  |  |  |  | 1.02 |  |  |  |  |  | 1.02 |
| *L. scabra* | CTT | 0.86 | L |  |  | 0.86 |  |  | 0.86 |  |  |  | 0.86 |  |  |
| *L. scabra* | GTG | 1.14 | V |  |  |  | 1.14 |  | 1.14 |  |  |  |  |  | 1.14 |
| *L. scabra* | ATT | 1.59 | I | 1.59 |  |  |  |  | 1.59 |  |  |  | 1.59 |  |  |
| *L. scabra* | ACA | 1.15 | T | 1.15 |  |  |  |  |  | 1.15 |  | 1.15 |  |  |  |
| *L. scabra* | CAT | 1.56 | H |  |  | 1.56 |  | 1.56 |  |  |  |  | 1.56 |  |  |
| *L. scabra* | ACG | 0.64 | T | 0.64 |  |  |  |  |  | 0.64 |  |  |  |  | 0.64 |
| *L. scabra* | GCA | 0.50 | A |  |  |  | 0.50 |  |  | 0.50 |  | 0.50 |  |  |  |
| *L. scabra* | TAT | 1.77 | Y |  | 1.77 |  |  | 1.77 |  |  |  |  | 1.77 |  |  |
| *L. scabra* | GGG | 2.06 | G |  |  |  | 2.06 |  |  |  | 2.06 |  |  |  | 2.06 |
| *L. scabra* | CCT | 1.47 | P |  |  | 1.47 |  |  |  | 1.47 |  |  | 1.47 |  |  |
| *L. scabra* | CGC | 0.40 | R |  |  | 0.40 |  |  |  |  | 0.40 |  |  | 0.40 |  |
| *L. scabra* | TCC | 0.69 | S |  | 0.69 |  |  |  |  | 0.69 |  |  |  | 0.69 |  |
| *L. scabra* | CTC | 0.16 | L |  |  | 0.16 |  |  | 0.16 |  |  |  |  | 0.16 |  |
| *L. scabra* | ACC | 0.67 | T | 0.67 |  |  |  |  |  | 0.67 |  |  |  | 0.67 |  |
| *L. scabra* | AGC | 0.54 | S | 0.54 |  |  |  |  |  |  | 0.54 |  |  | 0.54 |  |
| *L. scabra* | AAT | 1.38 | N | 1.38 |  |  |  | 1.38 |  |  |  |  | 1.38 |  |  |
| *L. scabra* | GTA | 0.58 | V |  |  |  | 0.58 |  | 0.58 |  |  | 0.58 |  |  |  |
| *L. scabra* | CAA | 1.42 | Q |  |  | 1.42 |  | 1.42 |  |  |  | 1.42 |  |  |  |
| *L. scabra* | TGG | 1.19 | W |  | 1.19 |  |  |  |  |  | 1.19 |  |  |  | 1.19 |
| *L. scabra* | GAG | 1.20 | E |  |  |  | 1.20 | 1.20 |  |  |  |  |  |  | 1.20 |
| *L. scabra* | TGT | 1.73 | C |  | 1.73 |  |  |  |  |  | 1.73 |  | 1.73 |  |  |
| *L. scabra* | AAC | 0.62 | N | 0.62 |  |  |  | 0.62 |  |  |  |  |  | 0.62 |  |
| *L. scabra* | AAG | 0.85 | K | 0.85 |  |  |  | 0.85 |  |  |  |  |  |  | 0.85 |
| *L. scabra* | GCC | 1.14 | A |  |  |  | 1.14 |  |  | 1.14 |  |  |  | 1.14 |  |
| *L. scabra* | CGA | 1.44 | R |  |  | 1.44 |  |  |  |  | 1.44 | 1.44 |  |  |  |
| *L. scabra* | GCT | 1.36 | A |  |  |  | 1.36 |  |  | 1.36 |  |  | 1.36 |  |  |
| *L. scabra* | CGT | 0.80 | R |  |  | 0.80 |  |  |  |  | 0.80 |  | 0.80 |  |  |
| *L. scabra* | TCG | 0.84 | S |  | 0.84 |  |  |  |  | 0.84 |  |  |  |  | 0.84 |
| *L. scabra* | CCA | 0.76 | P |  |  | 0.76 |  |  |  | 0.76 |  | 0.76 |  |  |  |
| *L. scabra* | TCA | 0.74 | S |  | 0.74 |  |  |  |  | 0.74 |  | 0.74 |  |  |  |
| *L. scabra* | GGA | 0.51 | G |  |  |  | 0.51 |  |  |  | 0.51 | 0.51 |  |  |  |
| *L. scabra* | CCG | 0.79 | P |  |  | 0.79 |  |  |  | 0.79 |  |  |  |  | 0.79 |
| *L. scabra* | GTC | 0.43 | V |  |  |  | 0.43 |  | 0.43 |  |  |  |  | 0.43 |  |
| *L. scabra* | TTC | 0.21 | F |  | 0.21 |  |  |  | 0.21 |  |  |  |  | 0.21 |  |
| *L. scabra* | CCC | 0.98 | P |  |  | 0.98 |  |  |  | 0.98 |  |  |  | 0.98 |  |
| *L. scabra* | AGT | 1.48 | S | 1.48 |  |  |  |  |  |  | 1.48 |  | 1.48 |  |  |
| *L. scabra* | GAA | 0.80 | E |  |  |  | 0.80 | 0.80 |  |  |  | 0.80 |  |  |  |
| *L. scabra* | AGA | 0.87 | S | 0.87 |  |  |  |  |  |  | 0.87 | 0.87 |  |  |  |
| *L. scabra* | CAC | 0.44 | H |  |  | 0.44 |  | 0.44 |  |  |  |  |  | 0.44 |  |
| *L. scabra* | CGG | 1.36 | R |  |  | 1.36 |  |  |  |  | 1.36 |  |  |  | 1.36 |
| *L. scabra* | CTG | 0.21 | L |  |  | 0.21 |  |  | 0.21 |  |  |  |  |  | 0.21 |
| *L. scabra* | TGC | 0.27 | C |  | 0.27 |  |  |  |  |  | 0.27 |  |  | 0.27 |  |
| **Average** | | | | **0.95** | **1.21** | **0.87** | **1.00** | **1.00** | **0.95** | **1.09** | **0.95** | **0.98** | **1.48** | **0.54** | **1.00** |
| **Average** | | | | **AT** | **1.08** | **CG** | **0.93** | **AT** | **0.97** | **CG** | **1.02** | **AT** | **1.22 ± 0.52** | **CG** | **0.77 ± 0.46** |

**Table S4. Long non-coding region: Microsatellite repeats**

| **Position** | **Motif** | **Repeats** | **Sequence** |
| --- | --- | --- | --- |
| 30 | 2 | 3 | TGTGTG |
| 53 | 2 | 3 | TTTTTT |
| 361 | 2 | 3 | TTTTTT |
| 747 | 2 | 3 | CTCTCT |
| 788 | 2 | 3 | GGGGGG |
| 837 | 2 | 5 | TTTTTTTTTT |
| 883 | 2 | 3 | TGTGTG |
| 915 | 2 | 3 | TGTGTG |
| 947 | 2 | 3 | TGTGTG |
| 1000 | 2 | 3 | GGGGGG |
| 1041 | 2 | 3 | GGGGGG |

**Table S5. Tandem Repeats Finder (Long non-coding region_1031bp):**

**Tandem Repeat 1:** Length: 1031: Found at i: 904 original size: 32 final size: 32

| **Position** | **Sequence** |
| --- | --- |
| 853 | TTTGTTTTAA |
| 863 | AGTTTTGTGTGGGAGGGGTCAAGGTTCCCTCG |
| 1 | AGTTTTGTGTGGGAGGGGTCAAGGTTCCCTCG |
| 895 | AGTTTTGTGTGGGAGGGGTCAAGGTTCCCTCG |
| 1 | AGTTTTGTGTGGGAGGGGTCAAGGTTCCCTCG |
| 927 | AGTTTTGTGTGGGAGGGGTTGGGGTCCCCTCG |
| 1 | AGTTTTGTGTGGGAGGGGTCAAGGTTCCCTCG |
| 959 | AGTTTTGTG |
| 1 | AGTTTTGTG |
| 968 | GAGGGGACTC |

Consensus pattern (32 bp):

AGTTTTGTGTGGGAGGGGTCAAGGTTCCCTCG

**Tandem Repeat 2.** Found at i: 998 original size:41 final size:41

| **Position** | **Sequence** |
| --- | --- |
| 937 | GGGAGGGGTT |
| 947 | GGGGTCCCCTCGAGTTTTGTGGAGGGGACTCCAAAGGAGGG |
| 1 | GGGGTCCCCTCGACTTTTGTGGAGGGGACCCCAAAGGAGGG |
| 988 | GGGGTCCCCTCGACTTTTGTGGAGGGGACCCCAAGGGAGGG |
| 1 | GGGGTCCCCTCGACTTTTGTGGAGGGGACCCCAAAGGAGGG |
| 1029 | GGG |
| 1 | GGG |

Consensus pattern (41 bp):

GGGGTCCCCTCGACTTTTGTGGAGGGGACCCCAAAGGAGGG

**Table S6. Shorter non-coding region: Microsatellite repeats**

| **Position** | **Motif** | **Repeats** | **Sequence** |
| --- | --- | --- | --- |
| 188 | 2 | 3 | GGGGGG |
| 203 | 2 | 3 | TTTTTT |
| 215 | 2 | 3 | TTTTTT |
| 274 | 2 | 4 | TTTTTTTT |
| 337 | 2 | 3 | GTGTGT |
| 343 | 3 | 3 | TTTTTTTTT |
| 551 | 2 | 3 | TTTTTT |

**Table S7. Tandem Repeats Finder (Shorter non-coding_591bp) :**

Found at i:367 original size:69 final size:69

| **Position** | **Sequence** |
| --- | --- |
| 242 | GGCCGTGGGT |
| 252 | TGGCTGGTACCCAA--G-TGGTAGTGTTTTTTTTCCTGAAGCATTTATGGGCGAGTTGTGGTTTG |
| 1 | TGGCTGGTACCCAAGTGATGGTAGTGTTTTTTTTCCTGAAGCATTTATGGGCGAGTTGTGGTTTG |
| 314 | AAAA |
| 66 | AAAA |
| 318 | TGGCTGGTACCCAAGTGATGGT-GTGTTTTTTTTTTCTGAAGCATTTATGGGCGGGTTGTGGTTT |
| 1 | TGGCTGGTACCCAAGTGATGGTAGTG-TTTTTTTTCCTGAAGCATTTATGGGCGAGTTGTGGTTT |
| 382 | GAAAA |
| 65 | GAAAA |
| 387 | TGGCTGGCACCCAAGTGAT |
| 1 | TGGCTGGTACCCAAGTGAT |
| 406 | AGTGATTCTT |

Consensus pattern (69 bp):

TGGCTGGTACCCAAGTGATGGTAGTGTTTTTTTTCCTGAAGCATTTATGGGCGAGTTGTGGTTTGAAAA

**
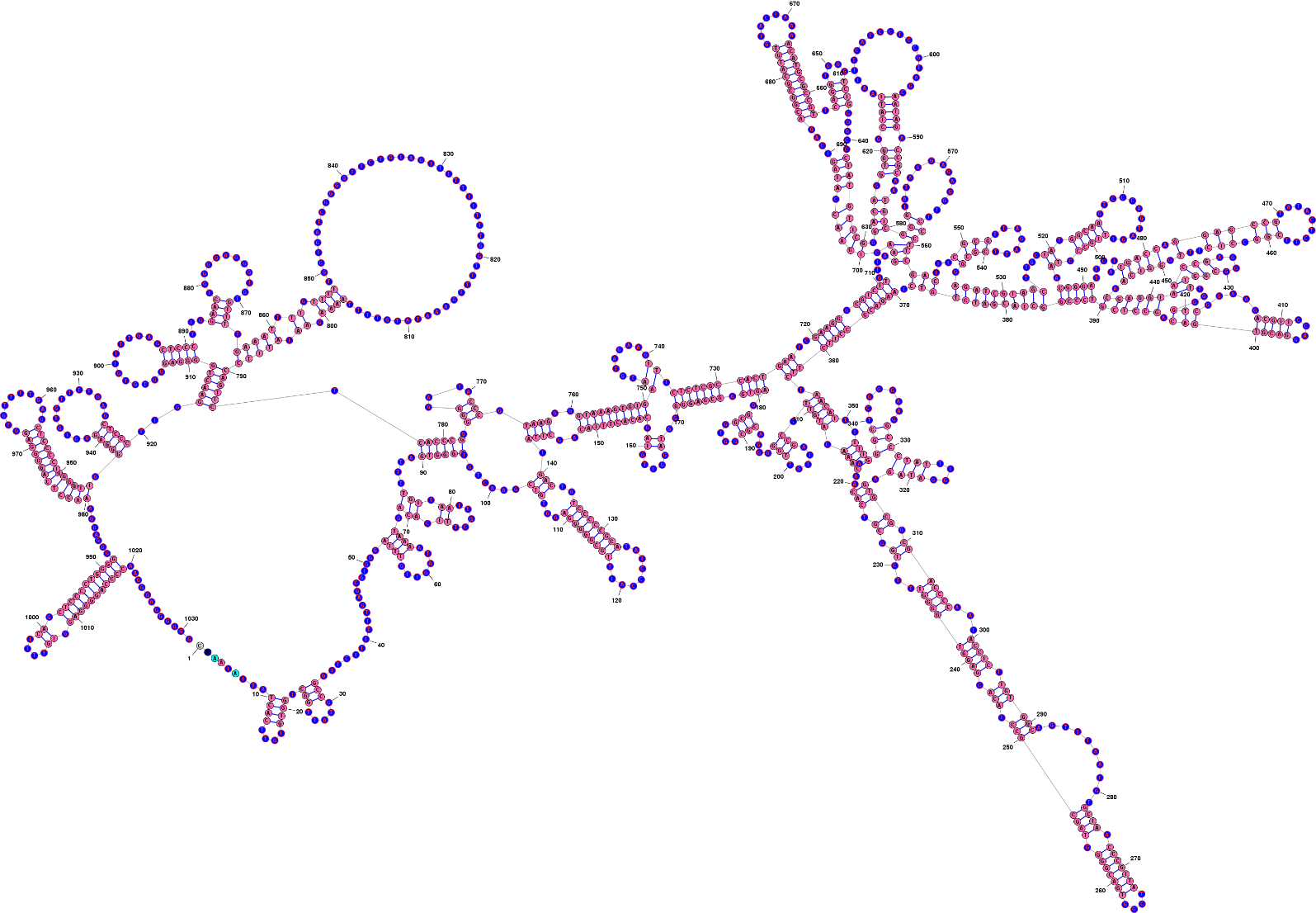
**

**Figure S1:** Secondary structure prediction (lowest free energy structure) in the long non-coding regions (1031 bp) present in the mitochondrial genome of *Leptoseris columna*


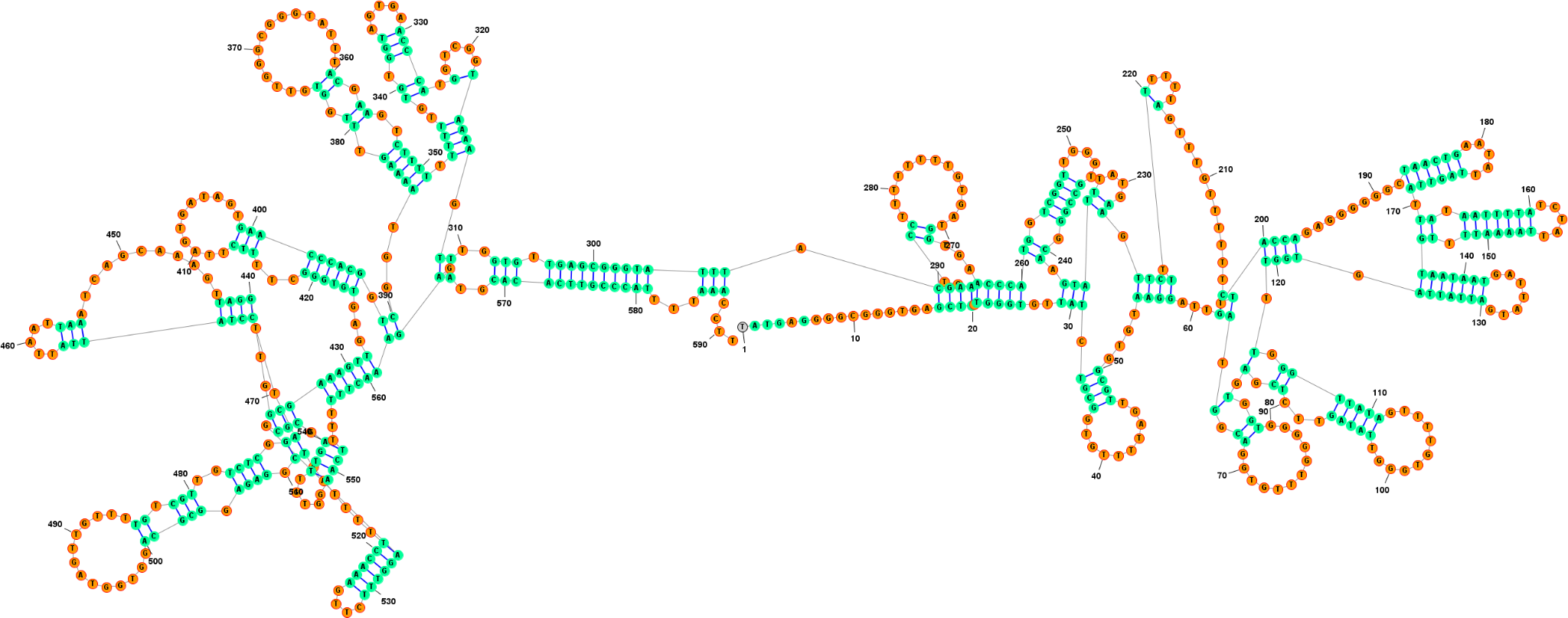


**Figure S2:** Secondary structure prediction (lowest free energy structure) in the shorter non-coding regions (591 bp) present in the mitochondrial genome of *Leptoseris columna*
